# Effectiveness of Art Therapy With Adult Clients in 2018—What Progress Has Been Made?

**DOI:** 10.3389/fpsyg.2018.01531

**Published:** 2018-08-29

**Authors:** Dafna Regev, Liat Cohen-Yatziv

**Affiliations:** School of Creative Arts Therapies, University of Haifa, Haifa, Israel

**Keywords:** art therapy, effectiveness evaluation, adult, systematic review, clinical populations

## Abstract

In the year 2000, an important art therapy literature review addressed an essential question—does art therapy work? It discussed 17 articles dealing with the issue of the effectiveness of art therapy. Two decades later, this research field has extended its scope and is flourishing. Several current reviews of research work have described the broad range of methods implemented today, which includes qualitative and quantitative studies; other reviews have focused on art therapy with specific populations, or by age group. The aim of this systematic literature review is to contribute to the ongoing discussion in the field by exploring the latest studies dealing with the effectiveness of art therapy with a broad scope of adult clients. We conducted a comprehensive search in four databases and review of every quantitative article that has addressed outcome measures in the art therapy field from 2000 to 2017. This paper presents the latest 27 studies in the field that examine the effectiveness of art therapy with adult clients and divides them into seven clinical categories: cancer patients, clients coping with a variety of medical conditions, mental health clients, clients coping with trauma, prison inmates, the elderly, and clients who have not been diagnosed with specific issues but face ongoing daily challenges. It underscores the potential effects of art therapy on these seven clinical populations, and recommends the necessary expansions for future research in the field, to enable art therapy research to take further strides forward.

In 1999, nearly two decades ago, the American Art Therapy Association (AATA) (1999) issued a mission statement that outlined the organization's commitment to research, defined the preferential topics for this research, and suggested future research directions in the field. One year later, Reynolds et al. (2000) published a review of studies that addressed the therapeutic effectiveness of art therapy. They included studies that differed in terms of research quality and standards. In eight studies by different authors, there was a single group with no control group; in four studies, there was a control group, but no randomization of the participants between the experimental group and the control group; and in only five studies was there randomization of the experimental group and the control group (RCT - Randomized Control Trial). They concluded that there was a substantial need to expand research in the field of art therapy to better determine the most appropriate interventions for different populations.

Two decades later, the field of research in art therapy has developed considerably. There are several reviews in the field that describe the expanding body of research work. Some of these reviews present studies that have examined the effectiveness of art therapy, without distinguishing between different populations. For example, as an extension of the work and review by Reynolds et al. (2000), Slayton et al. (2010) reviewed articles published between 1999 and 2007 that measured the outcome of art therapy sessions with different populations. Their review included qualitative studies, studies based on a single client in therapy, studies with no control groups, studies with a control group but with no randomization, and a small number of studies with a control group and randomization. They concluded that there has been progress in the field, but further research is needed. Four years later, Maujean et al. (2014) summarized high-quality studies that implemented RCT that focused on art therapy with adults. They found eight such studies that were conducted between 2008 and 2013. Seven reported beneficial effects of art therapy for adult clients, but they also concluded that more reliable controlled studies were needed to draw conclusions.

Together with these comprehensive reviews, many literature reviews have appeared in recent years discussing specific populations and a range of research methods. For example, in the field of art therapy for adults, Holmqvist and Persson (2012) overviewed art therapy studies on clients with psychosomatic disorders, eating disorders, or facing crises, based on case studies and intervention techniques. They concluded that there were not enough studies to prove that art therapy is effective for these specific disorders. Similarly, Geue et al. (2010) and a year later, Wood et al. (2011) examined art therapy with cancer patients. They assessed quantitative and qualitative studies and found that most studies have dealt with women suffering from breast cancer. They also documented the intervention techniques that were specifically used with this population, and reported that overall, the quantitative studies reported an improvement in a number of emotional domains faced by these clients. Another article by Huet (2015) reviewed articles dealing with ways to reduce stress in the workplace through art therapy intervention techniques. In this article, a total of 11 articles were discussed that employed different research methods. The authors focused on describing different ways to use art therapy in this context and argued that there has been a gradual emergence of a vast body of knowledge that reinforces the benefits of art therapy for people working in stressful work environments.

In the past three years, a number of literature reviews of controlled quantitative studies have dealt more specifically with the issue of the effectiveness of art therapy in treating specific populations. Schouten et al. (2015) overviewed quantitative studies in art therapy with adult trauma victims. They found that only six studies included a control group (only one of which included randomization) in this field. Half reported a significant reduction in trauma symptoms and another study found a decrease in the levels of depression in clients treated with art therapy. They pointed out that it is difficult to produce quantitative meta-analyses in art therapy given the limited size of the groups and because the evaluation is often based on several therapeutic methods that are used simultaneously. Further Uttley et al. (2015a,b) reviewed all the studies dealing with art therapy for adult clients with non-psychotic psychiatric disorders (anxiety, depression, and phobias). They found 15 randomized controlled quantitative studies of which 10 indicated that the therapeutic process was effective (positive changes following therapy in comparison to the control group). They were unable to conduct a meta-analysis due to the clinical heterogeneity and lack of sufficient information in the studies. In addition, they reviewed 12 qualitative studies that provided data on 188 clients and 16 therapists.

This article deals with research that focuses on measuring the effectiveness of art therapy. It addresses two major challenges. The first is the definition of the term “effectiveness.” We adopted the definition suggested in Hill et al. (1979); namely, “the attribute of an intervention or maneuver that results in more good than harm to those to whom it is offered” (p. 1203). The current review takes a positivist perspective (Holton, 1993) and relates to the measurement of effectiveness reported in quantitative studies that have been conducted in the field. Since the field of art therapy is still young, the scope of research is limited and the quality of research is diverse, which makes it difficult to create a comparative review that presents the knowledge in the field and draws thorough conclusions. Therefore, our review is based on the systematic review framework proposed in Case-Smith (2013) who divided the studies she reviewed into three levels of evidence. Level 1 refers to randomized controlled trials (RCT's), level 2 refers to nonrandomized two-group studies, and level 3 refers to nonrandomized one-group studies.

The second challenge has to do with the definition art therapy. We applied the standard definition provided by the American Art Therapy Association:

Art therapy, facilitated by a professional art therapist, effectively supports personal and relational treatment goals, as well as community concerns. Art therapy is used to improve cognitive and sensorimotor functions, foster self-esteem and self-awareness, cultivate emotional resilience, promote insight, enhance social skills, reduce and resolve conflicts and distress, and advance societal and ecological change (American Art Therapy Association, 2018).

This definition makes it clear that art therapy is a process that takes place in the presence of a certified art therapist, and indicates different areas where an effect or outcome in therapy can be expected as a result of this form of treatment.

Thus, the research question was formulated according to “PICOS” components (The PRISMA Group et al., 2009): Is art therapy effective for adult clients as measured in results published from 2000 to 2017, in various quantitative studies corresponding to Levels 1, 2, 3 (Case-Smith, 2013)? These studies assessed the effectiveness of art therapy on variety of indices including symptoms and physical measures, health or mental health assessments, quality of life assessment, or coping resources. These indices were typically evaluated through questionnaires and occasionally by projective drawings or physiological indices.

By posing this question, this systematic review joins the ongoing discussion in the field on the level of effectiveness of art therapy with adult clients. This forms part of the academization process in the field of art therapy, which involves attempting to relate intervention techniques in the field with their significance for theoretical research.

## Method

The search for relevant articles was carried out during the month of January 2017. Four major electronic databases were searched: Medline, PsycInfo, Scopus, and Web of Science. We searched for the term “art therapy” in the databases combined with the terms “Effectiveness,” “Efficacy,” “Outcome,” “Measurement,” “Treatment,” and “Intervention.” We restricted the search in the databases to articles published in English since the year 2000 for reasons of recency and the continued relevancy of the findings. In addition, all the literature reviews in the field (such as those reviewed above) were examined to locate additional articles that were pertinent to this study.

**Figure 1:**
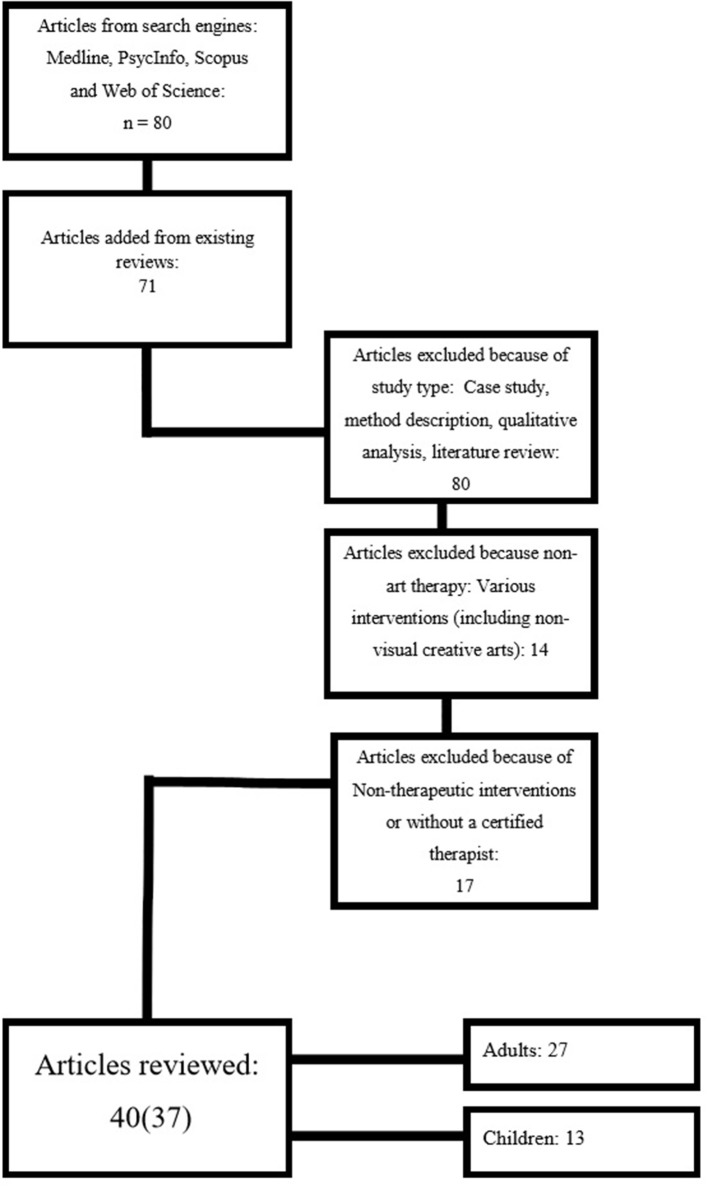
Search process.

During the initial screening stage, the abstracts were read by both authors (who are certified art therapists) to exclude those that were irrelevant to the purposes of the study. At this point 151 articles remained (see Figure 1).

In the next stage, the remaining articles were read and selected if they met the following inclusion criteria (see Figure 1):

- Reported a quantitative assessment of the effectiveness of art therapy on a sample of clients. Hence case studies, method descriptions, qualitative analyses, and literature reviews that did not meet these criteria were omitted. A total of 80 articles were removed at this stage.
- Enabled the assessment of the unique impact of art therapy. We thus omitted articles that described the use of a combination of therapeutic intervention techniques with a variety of art mediums simultaneously, not only visual art. A total of 14 articles were omitted at this stage.
- The art therapy was conducted in an ongoing manner in the presence of a certified art therapist. We thus omitted articles that described art intervention techniques that were not used in the context of therapy or were used in one-off art therapy interventions or therapy sessions with a non-certified art therapist. A total of 17 articles were removed at this stage.

Articles that met these inclusion criteria were defined as articles that examined the “effectiveness” of Art Therapy, and that quantified the impact of art therapy in a measurable way. A total of 37 studies were located in 40 articles (three studies were published in two different articles each). Of the 40 articles, 27 dealt with adult populations and are covered in this systematic review. This article categorizes mentioned articles in terms of the levels of evidence proposed by Case-Smith (2013).

## Findings

The findings derive from the 27 studies that we considered to have met the inclusion criteria. The choice to present the studies as a review rather than as a meta-analysis is due to the emergent nature of the field of art therapy. There is insufficient research in the field and the differences between studies and the indices measured are so great that it was impossible to produce a meta-analysis that would yield meaningful results (much like Uttley et al.'s conclusion, 2015a,b). In addition, the authors discussed the issue of the clinical categorization until full agreement was reached, to enable the reader to access the knowledge in the field in a way that will allow and encourage researchers to continue to conduct research. For samples where there has been more research (for example, art therapy with cancer patients), this area could have been separate and examined in and of itself, and relevant conclusions specific to this population could have been drawn. However, for other populations there was often a scarcity of studies which led us to group and categorize populations with similar characteristics (for example, medical conditions).

The next section presents the findings categorized into seven clinical categories. Different research methods were used: 17 of the articles (15 studies) used a comparison group with randomization (Level 1), five articles (four studies) used a comparison group without randomization (Level 2), and five articles used a single group without a comparison group (Level 3). In addition, there was a notable gender trend in that nine of the articles only examined women whereas only two of the articles exclusively referred to men. Sixteen did not define the research population by gender.

### Category 1: cancer patients

The first category consisted of art therapy with cancer patients (see Table 1). Six studies that examined effectiveness have been conducted with this specific population since 2006 and have been described in seven different articles (Monti et al., 2006, 2012; Oster et al., 2006; Öster et al., 2007; Bar-Sela et al., 2007; Svensk et al., 2009; Thyme et al., 2009). Five of the six studies were randomized (Level 1) and five dealt with women, most of whom had breast cancer. The total sample size ranged from 18 to 111 clients, most of whom were treated individually. Most of the therapeutic processes were short-term and ranged from five to eight sessions.

**Table 1:** Cancer patients.

**Article Title**	**Author (year)**	**Sample (size and groups)**	**Group Description**	**Intervention & Treatment**	**Amount and duration of therapy**	**Assessment Points**	**Outcome measured**	**Results**
A randomized, controlled trial of mindfulness-based art therapy (MBAT) for women with cancer	Monti et al. (2006)	N = 111 Randomly assigned to Mindfulness-based Art Therapy (MBAT) intervention (n = 56) Wait list control group (n = 55)	Women diagnosed with cancer. Each subject was between four months and two years of an original diagnosis of cancer (or remission). Patients were excluded if they were terminal, or had a current psychiatric diagnosis of a major mood disorder, psychotic disorder, or significant cognitive deficits as determined by their physicians.	MBAT - a supportive-expressive group therapy that includes skills training in mindfulness meditation and group art therapy tasks.	The intervention consisted of eight consecutive weekly meetings of two and a half-hours each i. Home assignments included the practice of mindfulness meditation 6 days a week for 30 min	Baseline and post-intervention (at weeks 8 and 16).	The Symptoms Checklist Revised (SCL-90-R), The Global Severity Index (GSI), The Medical Outcomes Study Short-Form Health Survey (SF-36).	As compared to the control group, the MBAT group demonstrated a significant decrease in symptoms of distress (as measured by the Symptoms Checklist-90-Revised) and significant improvements in key aspects of health-related quality of life (as measured by the Medical Outcomes Study Short-Form Health Survey). Level 1
Art therapy improves coping resources: A randomized, controlled study among women with breast cancer	Oster et al. (2006)	N = 41 Randomly assigned to Intervention group (n = 20) Control group (n = 21)	Women aged 37-69 (Median = 59) with non-metastatic primary breast cancer, referred to the Department of Oncology at Umeå University Hospital in Sweden for postoperative radiotherapy.	Individual art therapy intervention.	Five sessions, one hour each.	Baseline (start of radiotherapy) and 2 and 6 months after baseline.	Interviews, Diaries, Coping Resources Inventory (CRI).	There was an overall increase in coping resources among women with breast cancer after taking part in the art therapy intervention. Significant differences were found between the experimental and control groups in the social domain on the second and third occasions. Significant differences were also observed in the total score on the second occasion.
Art therapy for women with breast cancer: the therapeutic consequences of boundary strengthening	Öster et al. (2007)							The results showed a connection between participation in art therapy, talking about protecting one's own boundaries, and scoring higher on the CRI compared to the control group. There was also an association between the control group, repertoire conflicts, and lower scores on the CRI. Level 1
Art therapy improved depression and influenced fatigue levels in cancer patients on chemotherapy	Bar-Sela et al. (2007)	N = 60 Intervention group - patients who participated in 4 sessions or more (n = 19) participant group - patients who participated in 2 sessions or less (n = 41)	Cancer patients aged 25-72 (Median = 55) receiving chemotherapy.	The art therapists instructed each patient personally every week (Anthroposophical art therapy - painting with water-based paints). The sessions took place in a small room with eight workstations which was the maximum capacity for working with patients at the same time.	A variable amount of sessions - Once-weekly art therapy sessions. The patient chose how long to spend in the session, from a few minutes to more than an hour.	Before every session, relating to the previous week.	Hospital Anxiety and Depression Scale (HADS) and the Brief Fatigue Inventory (BFI).	BFI scores were significantly higher in the participant group. In the intervention group, the median HADS score for depression was 9 at the beginning and 7 after the fourth appointment (significant difference). The median BFI score went from 5.7 to 4.1 (Non- significant). The anxiety score was in the normal range from the beginning. Level 2
Art therapy improves experienced quality of life among women undergoing treatment for breast cancer: a randomized controlled study	Svensk et al. (2009)	N = 41 Randomly assigned to Intervention group (n = 20) Control group (n = 21)	Women –control- Median age = 55 Intervention -Median age = 59.5 undergoing radiotherapy treatment for breast cancer.	Individual art therapy sessions.	Five sessions, one hour each.	Baseline (start of radiotherapy) and 2 and 6 months after baseline.	WHOQOL-BREF and EORTC Quality of Life Questionnaire-BR23	The results indicated an overall improvement in QoL aspects among women in the intervention group. A significant increase in total health, total QoL, physical health and psychological health was observed in the art therapy group. A significant positive difference within the art therapy group was also seen concerning future perspectives, body image and systemic therapy side effects. Level 1
Individual brief art therapy can be helpful for women with breast cancer: A randomized controlled clinical study	Thyme et al. (2009)	N = 41 Randomly assigned to Intervention group (n = 20) Control group (n = 21)	Women aged 37-69 with breast cancer. Exclusion criterion was a preexisting physical or psychiatric illness.	The intervention in this study provided the participants with five individual sessions of art therapy where they were encouraged to express their feelings and thoughts.	Five sessions, one hour each.	Baseline (start of radiotherapy) and 2 and 6 months after baseline.	Structural Analysis of Social Behavior - The SASB, Symptom Check ist−90 The SCL90.	The hierarchical regression analyses suggested that art therapy was related to lower ratings of depression, anxiety, and somatic symptoms, as well as a lower level of general symptoms. Level 1
Changes in cerebral blood flow and anxiety associated with an 8-week mindfulness programme in women with breast cancer	Monti et al. (2012)	N = 18 Randomly assigned to Mindfulness-based Art Therapy (MBAT) (n = 8) Education control group (n = 10)	Women aged 52-77 who had been diagnosed with breast cancer between 6 months and 3 years prior to enrollment and were not in active treatment.	MBAT - a supportive-expressive group therapy that includes skills training in mindfulness meditation and group art therapy tasks.	Eight consecutive, weekly meetings of two and a half hours each in length.	Baseline, immediately after.	The fMRI imaging protocol consisted of five perfusion fMRI (using ASL) scans performed with a fixed order: ‘Resting 1, Neutral task (i.e. control), Meditation task (Body Scan), Stressor task, and Resting 2’. The response to the programme was evaluated utilizing the Symptom Checklist-90-Revised (SCL-90-R) as a behavioral rating.	Subjects in the MBAT group demonstrated significant increases in CBF (blood supply to the brain in a given period of time) at rest and during meditation in multiple limbic regions, including the left insula, right amygdala, right hippocampus and bilateral caudate. Patients in the MBAT programme also presented a significant correlation between increased CBF in the left caudate and decreased anxiety scores. In the MBAT group, responses to a stressful cue resulted in reduced activation of the posterior cingulate. The results indicated that the MBAT programme was associated with significant changes in CBF, which correlated with decreased anxiety over an 8-week period. Level 1

Some of the studies utilized different streams of art therapy. For example, the largest study of 111 participants, (Monti et al., 2006) included a mindfulness-based art therapy intervention—a combination of art therapy with mindfulness exercises. The measurement indices were very different for these studies and included questionnaires that examined physical symptoms, coping resources, quality of life, depression, anxiety, and fatigue. One specific study (Monti et al., 2012) also dealt with fMRI measurements. The findings of this category suggest that through relatively short-term interventions in art therapy (primarily individual therapy), it is possible to significantly improve the emotional state and perceived symptoms of these clients.

### Category 2: medical conditions

The second category consisted of art therapy with clients coping with a variety of medical conditions that were not cancer-related (see Table 2). Three studies examining the effectiveness of art therapy have been conducted since 2011, each of which deals with a completely different medical condition and employs a different research method. The earliest study dealt with art therapy with clients with advanced heart failure (Sela et al., 2011). This study had a sample size of 20 clients who were randomly divided into two groups (level 1). The clients participated in group art therapy for 6 weeks. A 2013 study addressed art therapy with clients coping with obesity (Sudres et al., 2013). This study examined 170 clients who were randomly divided into two groups (level 1). One group consisted of 96 clients who received art therapy for 2 weeks. A 2014 study addressed art therapy with 25 clients with HIV/AIDS (Feldman et al., 2014), who received art therapy in individual or group settings and did not include control groups (level 3). The duration of the therapeutic process was one or more sessions. Despite the considerable differences between the populations and the indices measured, these preliminary studies present an introductory description that points to the potential of art therapy to assist these populations.

**Table 2:** Medical conditions.

**Article Title**	**Author (year)**	**Sample (size and groups)**	**Group Description**	**Intervention & Treatment**	**Amount and duration of therapy**	**Assessment Points**	**Outcome measured**	**Results**
The influence of medical art therapy on quality of life and compliance of medical treatment of patients with advanced heart failure	Sela et al. (2011)	N = 20 Randomly assigned to Intervention group – Art Therapy (n = 10) Control group - routine clinical visit only (n = 10).	Patients with advanced heart failure.	A medical art therapist guided group A to express their feelings using art materials.	Met weekly for 8 weeks (First and last visits were individual, 6 group meetings).	Baseline, immediately after.	The Ulman, (a MAT diagnostic tool), the Minnesota Living with HF and compliance questionnaires.	Baseline Ulman, compliance and Minnesota scores were similar for the two groups. By the end of the study, the Ulman score improved significantly in the AT group compared to the control group as did the compliance score. In the AT group, the Minnesota score improved significantly in 7 patients and did not change in 3, while in the control group it improved in 2, did not change in 6 and worsened in 2. Level 1
Therapeutic patient education with art therapy: Effectiveness among obese patients	Sudres et al. (2013)	Randomly assigned to N = 170 AT (n = 74) Group 1 - without Group 2 - with AT (n = 96)	Obese patients. Group 1 – Mean age = 54.4 Group 2- Mean age = 54.5 The exclusion criteria were the following: diabetes, personality disorder diagnosis, presence of any antipsychotic or personality disorder requiring treatment, and the use of non-stabilized antidepressant or anti-anxiety treatment in the last 6 months.	Structured AT session workshops.	5 sessions, 2 hours each, over 2 weeks	Baseline, at the beginning of TPE program before the AT sessions (T0), after the two-week TPE program (T1) and at follow-up 6 weeks after the end of the TPE program (T2).	Torrance Tests of creative Thinking (TTCT), The Clinical Scale of Mediatised Therapies.	Significant weight loss was observed in both groups after 6 weeks following the TPE program. Group 2 subjects receiving art therapy showed an increase in quantitative indicators of creativity as well qualitative indicators as compared to Group 1 without AT. However, Group 1 without AT displayed a consistent reduction in all quantitative and qualitative indicators of creativity during and after the TPE program. Level 1
Process and Outcome Evaluation of an Art Therapy Program for People Living With HIV/AIDS	Feldman et al. (2014)	Only intervention group – (N = 25)	Adults living with HIV/AIDS – Mean age = 44.1. did not receive art therapy services prior to the baseline assessment.	Attended one or more individual or group art therapy sessions or open studio sessions.	One or more sessions.	Baseline and 6-month follow-up.	Depression Severity - The Patient Health Questionnaire (PHQ-9), The clients' health-related quality of life -The Short Form Health Survey (SF-12).	Statistically significant changes from baseline to 6-month follow-up in the desired direction were observed for both of the primary outcomes. Level 3

### Category 3: mental health

The third category covered art therapy with mental health clients (see Table 3). Four studies have been conducted since 2007 (two articles written on the same study—Crawford et al., 2012; Leurent et al., 2014, see Table 3). Research in this category falls into two main diagnostic areas. The first covers two studies on individuals with schizophrenia (Richardson et al., 2007; Crawford et al., 2012; Leurent et al., 2014) that involved randomization (level 1) with large samples (90-159 clients). The therapeutic process ranged from 12 sessions to a full year of therapy and included group therapy. The variety of indices that were used in these studies include measures of function, relationships and symptoms. Despite the attempt to use different types of research indices, in both studies, little or no effect was found to be associated with art therapy. Two studies were classified into the second diagnostic area: one addressing clients with psychiatric symptoms (Chandraiah et al., 2012) (level 3) and the other addressing women coping with depression (Thyme et al., 2007) (level 1). The therapeutic process ranged from 8 to 15 weeks. The findings reported in both studies suggested a change occurred in the duration of the therapeutic process. However, since neither study compared clients who received art therapy with those who received no therapy, it is difficult to evaluate the effectiveness of art therapy. Hence, the accumulated results of the studies in this category suggest that further research is needed to assess the effectiveness of interventions in art therapy for clients dealing with mental health issues.

**Table 3:** Mental health.

**Article Title**	**Author (year)**	**Sample (size and groups)**	**Group Description**	**Intervention & Treatment**	**Amount and duration of therapy**	**Assessment Points**	**Outcome measured**	**Results**
Exploratory RCT of art therapy as an adjunctive treatment in schizophrenia	Richardson et al. (2007)	N = 90 Randomly assigned to Intervention group – Art Therapy (n = 43) Control group - standard psychiatric care (n = 47)	Adults patients –Intervention group – Mean age = 39.6. Control group – Mean age = 42.6. Diagnosis of chronic schizophrenia of at least two years' duration and excluding those: (i) with organic illness, (ii) with a prior referral to AT services in the previous 2 years, (iii) currently receiving another formal psychological treatment, or (iv) currently admitted to inpatient care.	Group interactive art therapy was conducted according to the guidelines set out in Waller (1993, pp. 22 – 34). Through the availability and use of art materials and associated imagery the therapist promotes a climate in which the service user can learn about and understand those patterns of behavior which are causing distress.	12 weekly sessions of one and a half hours.	Baseline, immediately after, and at 6- month follow up.	General socio-demographic, clinical and health care utilization information, HONOS Scales rated in collaboration with the CPN, Brief Psychiatric Rating Scale (BPRS), Social Functioning Scale (SFS), Inventory of Interpersonal Problems (IIP-32), Scale for the Assessment of Negative Symptoms (SANS), Lancashire Quality of Life Profile (Perc QoL), Brief Symptom Inventory (BSI).	Art therapy produced a statistically significant positive effect on negative symptoms (assessed by Scale for the Assessment of Negative Symptoms) but had little and non-significant impact on other measures. Level 1
The outcome of short-term psychodynamic art therapy compared to short-term psychodynamic verbal therapy for depressed women	Thyme et al. (2007)	N = 39 Randomly assigned toArt psychotherapy (n = 18) Verbal psychotherapy (n = 21)	Women with depression – aged 19-53 (Mean = 33.8).	Individual art psychotherapy	AT group – average of 15 weeks. VT group - average of 20 weeks.	Baseline, immediately after,3-month follow-up.	The Impact of Event Scale (IES), The Symptom Check List 90 (SCL-90); Beck Depression Inventory (BDI); Hamilton Rating Scale of Depression (HRSD).	Participants in this study reported fewer depressive symptoms at the termination of psychotherapy compared to the initial level, and they reported even fewer symptoms at the 3-month follow-up. Observer-rated depressive symptoms showed a similar decline. The effect-sizes suggested a moderate to large change. The group variable did not contribute significantly to the analysis. Level 1
Efficacy of Group Art Therapy on Depressive Symptoms in Adult Heterogeneous Psychiatric Outpatients	Chandraiah et al. (2012)	Only intervention group – (N = 18) (Final sample - only 10 participants who attended 4 or more sessions)	Adult psychiatric outpatients aged 18-57 at a university medical center.	Group art therapy (6-8 in a group) - The beginning of each session was devoted to art making, usually 45–60 minutes, and the remaining 30 minutes was reserved for discussion.	8 sessions	Baseline, immediately after.	CES-D questionnaire - measures the level of depression experienced in the past week.	There was a statistically significant difference in the pre-treatment to post-treatment CES-D scores. Level 3
Group art therapy as an adjunctive treatment for people with schizophrenia: a randomized controlled trial (MATISSE) Moderating factors for the effectiveness of group art therapy for schizophrenia: secondary analysis of data from the MATISSE randomized controlled trial	Crawford et al. (2012) Leurent et al. (2014)	N = 159 Randomly assigned to Intervention – art therapy (n = 86) Control - activity groups attended at least one group (n = 73)	Adults aged 18 years or over (Mean = 41), had a clinical diagnosis of schizophrenia, confirmed by an examination of case notes.	Art therapy groups. Participants had up to eight members. Members were given access to a range of art materials and encouraged to use these to express themselves freely.	Weekly sessions of 90 min for an average period of 12 months.	Baseline, 12- and 24- month follow-up.	Completed by the researcher - Global functioning - using the GAF Scale, the Positive and Negative Syndrome (PANSS) Scale, Medication - using the Morisky Scale, the European Quality of Life-5 Dimensions (EQ-5D), the Adult Service Use Schedule (AD-SUS). Completed by the participant - the Social Function Questionnaire (SFQ), the General Well-Being Scale, the Client Satisfaction Questionnaire (CSQ). Completed by the participants' key worker - the four-item Service Engagement Scale (SES), Data on occupational and housing status, Any incidents of suicidal behavior, Global functioning - using the GAF Scale, details of any period of inpatient treatment. The Positive and Negative Syndrome Scale (PANSS), The Morisky scale, The Engagement and Acceptance Scale (EAS), Interview	No differences in primary outcomes (12 months) were found. Differences in secondary outcomes were not found, except that those referred to an activity group had fewer positive symptoms of schizophrenia at 24 months than those randomized to art therapy. The clinical effectiveness of group art therapy did not significantly differ between participants with more or less severe negative symptoms or between those who did and did not express a preference for art therapy. Level 1

### Category 4: trauma victims

The fourth category included art therapy with clients coping with trauma (see Table 4). In this category, two studies have been conducted since 2004, both with randomization (level 1). The first study (Pizarro, 2004) was composed of a sample of 45 students who participated in two art therapy sessions. These students had dealt with a traumatic event, which could occur at different levels of intensity and at various stages in their lives. In addition, the comparison was made between an art-therapy group and two comparison groups where one underwent writing therapy and the other experimented with artwork, regardless of the traumatic event. Despite the attempt to use a wide range of indices, including symptom reporting and emotional and health assessments, and perhaps because of the short duration of therapy, this study failed to find significant results.

**Table 4:** Trauma victims.

**Article Title**	**Author (year)**	**Sample (size and groups)**	**Group Description**	**Intervention & Treatment**	**Amount and duration of therapy**	**Assessment Points**	**Outcome measured**	**Results**
The Efficacy of Art and Writing Therapy: Increasing Positive Mental Health Outcomes and Participant Retention After Exposure to Traumatic Experience.	Pizarro (2004)	N = 45, Randomly assigned to Write-stress (n = 15) Art-stress (n = 15) Art-control (n = 15)	Undergraduate students. Write-stress – ages 18-20 (Mean = 18.47). Art-stress – ages 17-37 (Mean = 19.87). Art-control – ages 18-20 (Mean = 18.67).	Write/Art-stress - What I would like to have you write/draw about for the next two sessions is your most stressful or traumatic current or past experience. Art control - What I would like you to draw about over the next two sessions is your interpretation of this (photograph of a still life).	Two one-hour sessions were scheduled for each participant. The sessions were at least 1 day apart and at most 10 days apart.	Baseline, 1 month follow-up.	Demographic information, the General Health Questionnaire-28, the Global Measure of Perceived Stress, the Physical Symptoms Inventory, and the Shortened Version of the Profile of Mood States.	The write-stress condition presented a significant decrease in social dysfunction compared to the art-stress condition and to the art-control condition. Participants who completed artwork reported more enjoyment, were more likely to continue with the study, and were more likely to recommend the study to family and friends. The study was unable to demonstrate concrete health benefits from art therapy. Level 1
Humor, Self-Attitude, Emotions, and Cognitions in Group Art Therapy with War Veterans.	Kopytin and Lebedev (2013)	N = 112 Randomly assigned to Experimental group (n = 62) Control group (n = 50).	War Veterans aged 25-53 (Mean experimental group = 38; Mean control group = 35). The inclusion criteria were that participants had been diagnosed with having nonpsychotic mental disorders and had been involved in military campaigns. Patients were excluded from the study if they experienced severe mental disorders and were over 55.	Group sessions usually consisted of 5 to 8 patients. Each session was structured with warm-up activities, a main art- based activity with discussion, and closure.	Three times per week in after-lunch sessions that lasted 2.5 hours. The course of art therapy lasted one month and included 12 to 14 sessions.	Baseline, immediately after.	Symptomatic Checklists, SCL-90, Questionnaire of Depressive Conditions, the Integrative Anxiety Test, The self-report General Condition-Activity-Mood Test, The Silver Drawing Test (SDT) and Draw A Story assessment (DAS), The World Health Organization Quality of Life Questionnaire, The Humor scale.	When used as a brief intervention, group art therapy may exert a positive influence on war veterans and particularly on their symptomatic status, personality functioning, cognitive abilities and creativity and quality of life. Although these positive effects also were observed in the control group, they were less evident than in the experimental group. Scores on the DAS and SDT for emotional content, self-image, and cognition significantly increased for the experimental group after one month of art therapy; such increases were absent in the control group. Level 1

The second study (Kopytin and Lebedev, 2013) examined a sample of 112 war veterans who participated in 12–14 art therapy sessions. In this study, in which the definition of the traumatic event was more specific and defined by involvement in war, an attempt was also made to measure the level of improvement through a wide range of research indices, including reports of symptoms, emotional state, and quality of life. For some of the indices, there was a significant improvement compared to the control group.

These two articles thus present an inconsistent picture of the beneficial effects of this intervention, which may depend on the indices measured, the duration of therapy, and possibly the type of traumatic event.

### Category 5: prison inmates

The fifth category deals exclusively with David Gussak's extensive research on art therapy with prison inmates (Gussak, 2004, 2006, 2009a,b) (see Table 5). In this area three effectiveness studies have been conducted since 2004 (two articles were written on the same study; see Table 5). The first examined an intervention group without a control group (level 3), in contrast to the other two studies which did include control groups (level 2); the sample sizes ranged from 48 to 247 participants in the 2009 study. The art therapy intervention was carried out in a group setting and lasted 4 weeks in the first study to 15 weeks in the most recent study. Initially, Gussak used measurements solely from drawings (FEATS), but in later and more comprehensive research, depression and locus of control were also assessed. In the three studies, there was a reported improvement attributed to the art therapy intervention, as seen in the emotional state of the prison inmates.

**Table 5:** Prison inmates.

**Article Title**	**Author (year)**	**Sample (size and groups)**	**Group Description**	**Intervention & Treatment**	**Amount and duration of therapy**	**Assessment Points**	**Outcome measured**	**Results**
Art therapy with prison inmates: A pilot study.	Gussak (2004)	Only intervention group (N = 48)	Male inmates aged 21-63 medium- to maximum-security.	Six groups of eight members - art therapy interventions developed from simple to complex and from individual art tasks to more interactive group projects.	Twice a week for 4 weeks.	Baseline, immediately after.	The Draw a Person Picking an Apple from a Tree evaluation - (FEATS), Survey - developed specifically for the pilot study by the primary investigator - seven categories focusing on the inmate's interactions and compliance with prison rules and expectations.	There was significant change in seven of the 14 scales of FEATS: Prominence of Color, Color Fit, Implied Energy, Space, Integration, Details of Objects, and environment and Line Quality. No results regarding the survey. Level 3
The effects of art therapy with prison inmates: A follow-up study.	Gussak (2006) -	N = 44 Intervention group (n = 27) Control group (n = 17)	Male Inmates aged 21 to 59.	Four groups - art therapy interventions developed from simple to complex and from individual art tasks to more interactive group projects.	Once a week for 8 weeks.	Baseline, immediately after.	The Draw a Person Picking an Apple from a Tree evaluation - (FEATS), The Beck Depression Inventory–Short Form (BDI-II).	BDI-II - The experimental group had significantly greater decrease from pretest to posttest than the control group. FEATS - The experimental group's rotation was greater than the control group's rotation. Level 2
The effects of art therapy on male and female inmates: Advancing the research base. Comparing the effectiveness of art therapy on depression and locus of control of male and female inmates.	Gussak (2009a) Gussak (2009b)	N = 247 Intervention group (n = 98 women + 75 men). Control group (n = 29 women + 45 men)	Inmates Intervention group – women – ages 25-51. Intervention group – men – ages 22-50. Control group – women – ages 20-47 Control group – men – ages 24-51. Medium to maximum adult correctional facilities.	Groups - art therapy interventions developed from simple to complex and from individual art tasks to more interactive group projects.	One session period lasted 15 weeks, one session per week.	Baseline, immediately after.	The Beck Depression Inventory-Short Form (BDI-II), the Adult Nowicki-Strickland Locus of Control Scale (ANS), The Draw a Person Picking an Apple from a Tree evaluation - (FEATS)	Overall, the results of the BDI-II and the ANS supported the hypotheses, while the FEATS did not. The results indicated a trend toward significance in a greater improvement in mood and internal locus of control in female inmates than male inmates. Level 2

### Category 6: the elderly

The sixth category covered art therapy with the elderly (see Table 6). Three effectiveness studies have been conducted since 2006: one study was conducted with healthy Korean American older individuals (Kim, 2013), the second study involved older individuals coping with depression (McCaffrey et al., 2011), and the third dealt with older individuals with moderate to severe dementia (Rusted et al., 2006). In all three studies, the participants were randomly divided into groups (level 1), in a group therapy setting, with a sample size of 39–50 clients. The number of sessions ranged from 6 to 40. The authors of these studies were interested in a variety of indices. In both the study of elderly Koreans and the elderly coping with depression, various aspects of the emotional state of the clients were measured. Art therapy was considered to have led to an improvement on these measures. In a study of older people with dementia, many observational measures were used to assess emotional states, behavior, and abilities, but change was found only in some of them.

**Table 6:** The Elderly.

**Article Title**	**Author (year)**	**Sample (size and groups)**	**Group Description**	**Intervention & Treatment**	**Amount and duration of therapy**	**Assessment Points**	**Outcome measured**	**Results**
A Multi-center Randomized Control Group Trial on the Use of Art Therapy for Older People with Dementia	Rusted et al. (2006)	N = 45 Randomly assigned to art therapy or activity groups.	Patients Women – ages 74-92 (Mean = 84.05) Men – ages 67-92 (Mean-80.33) diagnosed with mild to severe dementia. Inclusion criteria were diagnosis of dementia (mixed origin), attendance at day care or residential facility, previous diagnosis by consultant psychogeriatrician, confirmatory diagnosis from medical records. Exclusion criteria were additional psychiatric disorders.	Art therapy or activity groups (with a maximum of six participants per group). For the art therapy groups, a group-interactive, psychodynamic approach was employed.	One hour each week for 40 successive weeks.	Six assessment points - (at baseline, ten, 20 and 40 weeks into group work, with one and three months follow-up).	Cornell Scale for Depression in Dementia (CSDD), The Multi Observational Scale for the Elderly (MOSES), The Mini-Mental State Exam (MMSE), The Rivermead Behavioral Memory Test (RBMT), Tests of Everyday Attention (TEA), Benton Fluency Task, Bond-Lader Mood Scale, Skill Builders, Clifton Assessment Procedures for the Elderley (CAPE), the Rating Scale for Aggressive Behaviour in the Elderly.	This research provided clear evidence of positive and durable benefits in aspects of mental alertness, sociability, physical and social engagement in clients with moderate and severe dementia. These changes were quantitatively different from the pattern of effects achieved with a parallel programme of recreational activity. Level 1
Garden walking and art therapy for depression in older adults: a pilot study	McCaffrey et al. (2011)	N = 39 Randomly assigned to Art Therapy (n = 13) Group Walking/ Guided Imagery (n = 13) Independent Walking (n = 13)	Art Therapy – Mean age = 74.30 (S.D. = 6.4) Group Walking/ Guided Imagery Mean age = 74.60 (S.D. = 4.98) Independent Walking – Mean age 73.90 (S.D. = 6.79) Inclu-sion criteria were that participants had self-diagnosed or health care provider-diagnosed depression, were able to walk approximately 1 mile, were older than 65, and could get to the gardens twice per week for 6 weeks.	The art therapy group met with a certified art therapist. This group began by drawing a self-portrait and presenting their portrait to the entire group. New drawings and discussions took place each week. The independent and guided garden walking groups met on different days at the gardens. Participants in the walk alone group signed in and walked the garden alone.	6 weeks. The art therapy group met twice per week.	Baseline, immediately after.	Geriatric Depression Scale (GDS), *Positive- and Negative-Emotion Word Use*.	Significant decreases were found in depression for all three groups from pretest to posttest. No significant differences were noted between the groups over time. Pos-itive-emotion word use increased and negative-emotion word use decreased. Regardless of intervention group, groups did not differ over time. Level 1
A randomized, controlled study of the effects of art therapy on older Korean-Americans' healthy aging	Kim (2013)	N = 50 Randomly assigned to group A, the art therapy intervention group (AG) (n = 21 women and 4 men). Group B, the control group (CG) (n = 18 women and 7 men) Later, assigning them to each group (AG or CG) alternately.	Korean American older individuals – AG- ages 69-87 (Mean = 77.64, S.D. = 5.51) CG – ages 72-86 (Mean = 78.76, S.D. = 4.02). From two adult day health care programs (ADHCP)	The structure of the art therapy intervention was based on the psycho-cybernetics approach to art therapy (Nucho, 2003). Each session consisted of 10–15 min for introduction as an “unfreezing” phase, 35–40 min for individual art-making as a “doing and dialoging” phase, and 15–20 min for group discussion as an “ending and integrating” phase	AG - 4 weeks of art therapy at a frequency of three times per week (for a maximum of 12 sessions). The total session time was 60–75 min.	Baseline, immediately after.	The Positive and Negative Affect Schedule (PANAS), the State-Trait Anxiety Inventory (STAI), the Rosenberg Self-Esteem Scale (RSES).	Participants in the AG showed a greater change on the PANAS in a positive direction compared to the CG; Participants in the AG reported a greater decrease in both state and trait anxiety after the art therapy intervention compared to those in the CG; Participants in the AG showed a greater increase in self-esteem after the intervention compared to those in the CG; Level 1

The findings suggest that art therapy seems to have a beneficial effect on older individuals who are coping with a variety of challenges in their lives, as reflected in the changes in the indices in these studies.

### Category 7: clients who face ongoing daily challenges

The seventh category consisted of art therapy with clients who face ongoing daily challenges that do not fall into one diagnostic category (see Table 7). Three studies have been conducted since 2008, two of which address issues such as stress, distress, and burnout of individuals working in various health professions (Italia et al., 2008; Visnola et al., 2010). These studies were carried out without randomization; in one study (Visnola et al., 2010) there was a control group (level 2), whereas in the other (Italia et al., 2008) there was not (level 3). The sample size ranged from 20 to 60 participants. The therapeutic process lasted 9–13 sessions in a group art therapy setting. These studies suggest that art therapy can help healthcare professionals reduce levels of stress, anxiety, and burnout connected to their work.

**Table 7:** Clients who face ongoing daily challenges.

**Article Title**	**Author (year)**	**Sample (size and groups)**	**Group Description**	**Intervention & Treatment**	**Amount and duration of therapy**	**Assessment Points**	**Outcome measured**	**Results**
Evaluation and art therapy treatment of the burnout syndrome in oncology units.	Italia et al. (2008).	Only intervention group (N = 20)	Doctors and nurses from the Regional Reference Center for Pediatric Oncology at the University General Hospital.	Group meetings with the aim of increasing collaboration and relationships among the members of the curing team using the creative techniques of art therapy as a form of supportive and not psycho-therapeutic action.	The program was delivered 13 weekly meetings.	Baseline, immediately after.	The Maslach Burnout Inventory.	Comparing the responses from participants before and after the intervention indicated a statistically significant decreased level of burnout. Level 3
Effects of art therapy on stress and anxiety of employees	Visnola et al. (2010)	N = 60 Intervention group (n = 30) Control group (n = 30)	Health care workers (women), ages 20-69.	The intervention group participated in an art therapy program consisting of three stages: 1) situation determination; 2) acquiring of methods of stress reduction and overcoming of anxiety; 3) awareness of self-conception and strengthening of potential (Visnola, 2009). The sessions were structured.	Nine sessions in total, 18 hours in two months.	Baseline, immediately after.	The Stress Questionnaire, the Spielberger examination of anxiety with State-trait Anxiety Inventory Form Y−1, the high performance liquid chromatography method (HPLC Water Alliance with UV detection) to establish levels of cortisol in saliva.	Before and after art therapy in the experimental group, the level of the stress indicator (cortisol) over twenty-four hours and also the state of anxiety decreased significantly. The mean final stress level and situational anxiety for this group were significantly lower than for the control group. No significant changes were found for trait anxiety between groups. Level 2
A pilot study assessing art therapy as a mental health intervention for subfertile women	Hughes and da Silva (2011)	Only intervention group (N = 21)	Women (Mean age = 35.7, S.D. = 2.1) attending the Hamilton Health Sciences fertility clinic for ongoing fertility care.	The eight group sessions were semi-structured and employed a different art therapy technique each week. Four to seven women per group.	Two-hour art therapy sessions once weekly, for 8 weeks.	Baseline, immediately after.	the Beck Anxiety Inventory, Beck Depression Inventory-II and Beck Hopelessness Scale	Clinically and statistically significant reductions were seen in Beck Depression Inventory-II Scale and Beck Hopelessness Scale, while the change in Beck Anxiety score was not statistically significant. Level 3

The third article addresses art therapy for women undergoing fertility treatment (Hughes and da Silva, 2011). The sample only included an intervention group (level 3) consisting of 21 women in a group art therapy setting. This study reported a reduction in anxiety and in feelings of hopelessness. The samples in the studies in this category were relatively small and usually did not include a control group. However, there is potential for further research in this area.

## Discussion and conclusion

The purpose of this review was to assess whether art therapy is effective for adult clients as measured in quantitative studies published from 2000 to 2017. Notably, since the Reynolds et al. (2000) review, the body of knowledge in this field has grown and established itself significantly, and a growing number of RCT studies (level 1) have been conducted with larger sample sizes. The advantage of such studies lies in the lesser likelihood of Type I errors as opposed to other studies with no control group or studies that have a control group but no randomization. Nevertheless, there are still only a small number of studies addressing each population, and these studies differ considerably in terms of the course of the therapeutic process, the proposed interventions and the indices that were examined, hence making a meaningful meta-analysis impossible. The findings however are largely encouraging and show a growing trend toward conducting more carefully designed studies that lend themselves to validation and replication; yet—there is a long road ahead. In the past, the effectiveness of art therapy was noticeable to those involved in the field, but less to other professionals. Today, by contrast, there are impressive published findings in a variety of areas. These studies can help expand the contribution of art therapists in other areas and with other populations.

During our search, we were struck by the large number of articles which appear to present interventions in the field of art therapy, but in fact were conducted by non-certified art therapists or were restricted to a therapeutic intervention of a single session in a manner that would not be considered therapy. The existence of such studies emphasizes the continued need to define, clarify and specify what art therapy is and what it is not, and specifically to clarify that this type of therapy must be composed of ongoing sessions and be conducted by a certified art therapist who meets the criteria defined for the profession (American Art Therapy Association, 2018).

The first two clinical categories dealt with clients who are coping with a variety of medical conditions. In this section, we were surprised by the vast amount of research in the field of art therapy with cancer patients, most of which were categorized as level 1. Art therapy emerges strongly as a way to enhance their quality of life and their ability to cope with a variety of psychological symptoms. Our review supplements previous reviews in the field (Geue et al., 2010; Wood et al., 2011) and shows that the findings on art therapy with cancer patients are primarily based on higher levels of evidence studies with randomization and relatively large samples.

The second category, which dealt with clients with a range of medical problems, was intended primarily to list the preliminary research in this field, due to the wide variability between the different populations. The differences in the populations treated suggests that, the measurement tools should be adapted to each type of medical issue. The only instrument that could possibly be applied to all these populations in future research is one that measures improvement in quality of life. It is surprising to note that unlike research on cancer patients, which has been considerable, there have been few studies on individuals with other medical conditions.

The third category dealt with clients with mental health issues. In this category we focused solely on adult clients (as opposed to children which will be reviewed in a separate article) and differentiated from the elderly (category 6). In addition, they were separated from clients coping with trauma (category 4). As a result, a relatively small number of studies met the strict criteria of this review regarding what could be defined as art therapy for clients with mental health issues, although some of the studies had large sample sizes and showed a higher level of evidence. For clients coping with schizophrenia, the reviewed findings are not optimistic. These data are congruent with the many articles on psychotherapy that have addressed this population and have emphasized the complexity of treating such individuals (Pfammatter et al., 2006). Studies have shown that the most effective therapeutic approach for this population appears to be cognitive-behavioral (Turner et al., 2014). Thus, future work should examine the effectiveness of the cognitive-behavioral approach in art therapy for this population. More research is also needed to better understand how art therapy can be effective with clients experiencing other mental health issues.

The fourth category addressed clients coping with trauma. While there have been few studies in this field, all of them are in a higher level of evidence. It is important to note that these studies did not assess post-traumatic stress disorder (PTSD), but rather individuals who have dealt with traumatic events. Even though the first study (Pizarro, 2004) did not confirm the effectiveness of art therapy, the limited number of sessions with each client may have been a major factor. When dealing with trauma, there is a need for thorough processing of the experience, and it is quite possible that two sessions were insufficient. The second study (Kopytin and Lebedev, 2013) reported that art therapy was beneficial when the intervention lasted longer. These data are consistent with the Schouten et al. (2015) review. Certain studies reviewed by Schouten et al. (2015) were not mentioned in our review because some were not published as articles, and others included single session interventions that were not led by a certified art therapist.

The fifth category addressed prison inmates. In this field, it is worth mentioning the work of Gussak, a researcher who has studied the field and conducted several studies with an increasing number of participants. His findings undoubtedly point to the potential of art therapy for inmates particularly in long term interventions.

The sixth category addressed the elderly. The field of geriatric art therapy has been gaining momentum in recent years (Im and Lee, 2014; Wang and Li, 2016). It is clear from the articles that group therapy sessions are particularly suitable for these clients and that it is important to continue conducting research to target effective intervention methods for this population. The research findings certainly indicate the potential of this field.

The seventh and final category dealt with clients who are facing daily challenges in their lives. The findings suggest that art therapy can be a suitable form of treatment and a way to mitigate issues such as stress and burnout at work.

Overall, this review documents the extensive research conducted in recent years; although qualitative studies were not included in this article, there is no doubt that using a variety of research methods can help expand knowledge in the field. As concerns quantitative studies, the review examined the effectiveness of art therapy for adult clients from research in the field from recent years and with reference to seven clinical categories.

The current review has several limitations. First, due to the small number of studies in the field, it includes various levels of quantitative studies. Some lack comparison groups and others include comparison groups with other treatment methods (for example verbal therapy). This variability makes it difficult to generalize across findings, but not mentioning these studies would have led to the inclusion of an even smaller number of studies. Second, in many studies there are several indices of varying types (questionnaires, drawings, physiological indices). Occasionally, only some of these indices led to demonstrable indications of the effectiveness of art therapy. Due to the complexity of the findings, we were not always able to detail these subtleties and challenges in the current review, and future researchers interested in the field should examine these specific studies closely before conducting further research on the same population. In addition, due to the limited number of studies in this field, we needed to combine various subjects in certain cases, make decisions, and create artificial categories based on our professional knowledge and judgment. For example, the article on female infertility (Hughes and da Silva, 2011) was placed in the seventh category of ongoing and daily challenges, and not in the second category of medical problems, due to the feasibility of this condition for various reasons, which are not necessarily medical.

Research in the field can be expanded in several ways. First, art therapy is a very broad domain that covers diverse populations, some of which have not yet been studied at all in the context of treatment effectiveness. Second, based on the conclusions derived from this review future studies should be planned so that they are performed by a certified art therapist, over a continuous period of time and on large enough samples. In so doing, within approximately a decade, it should be possible to produce a meaningful meta-analysis based on significant and comparable findings from the field, which could lead to more advanced and specific conclusions. Third, in order to raise the level of research in our field, it is important for researchers to devote time and thought to planning studies at the highest level (level 1). Large samples are not enough; one should also consider well-controlled studies (RCT), the blindness of the experiment, the blindness of the participants and the experimenters to the purpose of the research, the division of research groups and so on (Liebherz et al., 2016; Munder and Barth, 2018). Finally, it is of great importance that researchers will select valid and reliable research tools that have been used extensively.

This documentation of the numerous studies on the effectiveness of art therapy was long and complex, but also filled us with hope. We are optimistic that this article will take the field one step further in this direction.

## Author contributions

All authors listed have made a substantial, direct and intellectual contribution to the work, and approved it for publication.

### Conflict of interest statement

The authors declare that the research was conducted in the absence of any commercial or financial relationships that could be construed as a potential conflict of interest.
